# Revision of the wingless *Sikkimia* Duvivier (Coleoptera, Chrysomelidae, Galerucinae) from Taiwan, including a new generic synonymy and four new species descriptions

**DOI:** 10.3897/zookeys.553.6576

**Published:** 2016-01-14

**Authors:** Chi-Feng Lee, Jan Bezděk

**Affiliations:** 1 Applied Zoology Division, Taiwan Agricultural Research Institute, 189 Chung-Cheng Road, Wufeng, Taichung 413, Taiwan; 2 Department of Zoology, Mendel University, Zemědělská 1, 613 00 Brno, Czech Republic

**Keywords:** Leaf beetles, Polygonum chinense, nocturnal behavior, taxonomic revision

## Abstract

The genus *Taiwanolepta* Kimoto, 1989 (type species *Taiwanolepta babai* Kimoto, 1989) is proposed as a junior synonym of *Sikkimia* Duvivier, 1891. *Sikkimia* species from Taiwan form a group characterized by the reduction of their hind wings. Most of them cannot be distinguished using external morphology, except by the structure of last two antennomeres in males. Diagnoses are made by using distribution, aedeagal, and gonocoxal morphology. The group includes one previously described species, *Sikkimia babai* (Kimoto, 1989), **comb. n.**, and four new species, *Sikkimia meihuai* **sp. n.**, *Sikkimia sufangae* **sp. n.**, *Sikkimia tsoui* **sp. n.**, and *Sikkimia yuae* **sp. n.** Speciation models, supporting the high diversity of *Sikkimia* species in Taiwan, are discussed. *Sikkimia metallica* Jacoby, 1903 and *Sikkimia tamra* Maulik, 1936, both from southern India, are transferred to the genus *Cerophysa* Chevrolat, 1836.

## Introduction

Subsequent to the original description of the genus, several new genera have been proposed for *Sikkimia* species. Based on the study of type specimens, the genera *Yunomela* Chen, 1964 and *Vietocerus* Lopatin, 2003 were synonymized with *Sikkimia* by Bezděk and Zhang (2006). Another genus, *Taiwanolepta* Kimoto, 1989, is here synonymized. While continental *Sikkimia* species have well developed wings and are capable of flying, the Taiwanese species have, until now, been classified in *Taiwanolepta* and are wingless and nocturnal. In Taiwan, *Sikkimia* species appeared to be rare as no recent records had been reported.

The basic bionomics of Taiwanese *Sikkimia* populations can be summarized as follows: adults are nocturnal and closely associated with these host plants: *Polygonum chinense* L., *Polygonum posumbu* Buch.-Ham. ex Don, and *Polygonum thunbergii* Sieb. & Zucc. (Polygonaceae); *Rubus swinhoei* Hance and *Rubus corchorifolius* L. f. (Rosaceae); and Dumasia miaoliensis Y. C. Liu & F. Y. Lu subsp. bicolor (Hayata) Ohashi & Tateishi (Fabaceae); mainly feed on the host plant *Polygonum chinense*. This plant is widely distributed and grows on the edges of forests, roads, walking trails, and rivers. As these environments are easily accessible, adults can be collected by searching for adults on host plants at night. Approximately 350 specimens have been collected throughout Taiwan by members of the Taiwan Chrysomelid Research Team (TCRT) led by author Lee.

## Materials and methods

Larvae were put into small glass containers (diameter 142 mm × height 50 mm) with cuttings from their host plants at average 20.8 °C, 74%RH, with a photoperiod of 12:12 (L:D) for laboratory rearing. When mature larvae began searching for pupation sites, they were transferred to smaller plastic containers (diameter 90 mm × height 57 mm) filled with moist soil (about 80% of container volume).

The abdomen was separated from the body and boiled in a 10% KOH solution, followed by washing in distilled water to prepare genitalia for drawing purposes. The genitalia were then dissected from the abdomen, mounted on slides in glycerin, and studied and drawn using a Leica M165 stereomicroscope. For detailed examination a Nikon ECLIPSE 50i microscope was used.

At least three pairs from each species were examined to delimit the variability of diagnostic characters,. When a species was collected from more than one locality, at least one pair from each locality was examined. Females are associated with a distinct species based on localities where they were collected. Length is measured from the anterior margin of the eye to the elytral apex, and width at the greatest width of the elytra.

Specimens studied herein are deposited at the following institutes: The Natural History Museum (BMNH), London, UK; Jan Bezděk collection (JBCB), Brno, Czech Republic; Ehime University (EUMJ), Matsuyama, Japan; Kitakyushu Museum of Natural History and Human History (KMNH), Kitakyushu, Japan; TARI: Taiwan Agricultural Research Institute, Taichung, Taiwan. Depositions are indicated with their recognized abbreviations except for those deposited at TARI.

## Taxonomy

### Genus Sikkimia

Taxon classificationAnimaliaColeopteraChrysomelidae

Duvivier, 1891

Sikkimia Duvivier, 1891: 154 (type species: *Sikkimia antennana* Duvivier, 1891, by monotypy); Maulik 1936: 520 (redescription).
Yunomela Chen, 1964: 201 (type species: *Yunomela rufa* Chen, 1964, by original designation); Bezděk and Zhang 2006 (as synonym of *Sikkimia*).
Taiwanolepta Kimoto, 1989: 73 (type species: *Taiwanolepta babai* Kimoto, 1989, by original designation). **New Synonym**
Vietocerus Lopatin, 2003: 103 (type species: *Vietocerus kabakovi* Lopatin, 2003, by original designation); Bezděk and Zhang 2006 (as synonym of *Sikkimia*).

#### Remarks.

**Figures 1–2.**
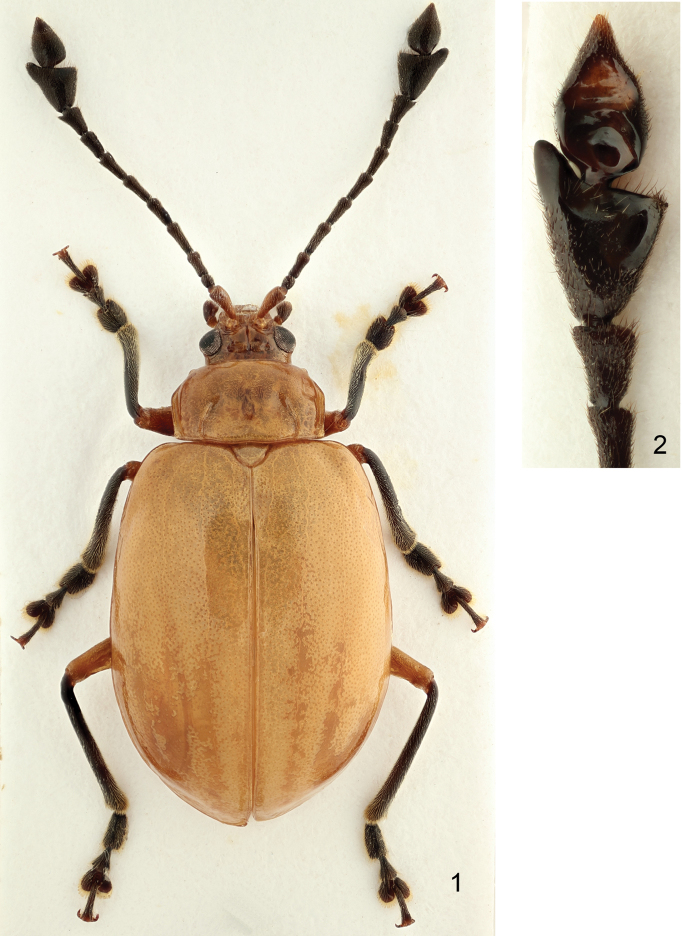
*Sikkimia rufa* (Chen). **1** Male, dorsal view **2** Three apical antennomeres of right antenna, ventral view.

The diagnostic characters for the genus *Sikkimia*, as indicated by Bezděk and Zhang (2006), are modified and extended as follows: body large (6.1–12.0 mm), robust, orange, red or brown; last two segments of antennae strongly enlarged in most males (Figs 1–2); frontal tubercles large, sub-quadratic; pronotum with antebasal transverse impression, limited on sides by short longitudinal furrows, and an additional longitudinal groove half way between short longitudinal furrows and lateral margin, running parallel to the lateral margin (Fig. 1); all pronotal margins bordered; procoxal cavities closed; apical ventrite trilobed in male, with internal anterior margin extended (Figs 7, 48 & 49); and claws appendiculate.

As all the main diagnostic characters are shared by both the continental and Taiwanese species, *Taiwanolepta* is here synonymized with *Sikkimia*. Taiwanese species differ from the continental species in having a shorter body (6.1–9.0 mm) reduced hind wings and consequently reduced humeral calli. The apical antennomere in the male is spear-shaped and more or less symmetrical in continental species (Fig. 2, see also the drawings in Bezděk and Zhang (2006)), while strongly asymmetrical in Taiwanese species (Figs 24–47). Outer longitudinal grooves on pronotum are deeper in Taiwanese species while more feeble in continental species. Aedeagus sclerotized ventrally in Taiwanese species, but membranous in continental species. Internal sclerite divergent apically in almost all Taiwanese species (Figs 19, 53, 66, 86), while the continental *Sikkimia rufa* has the sclerite divergent basally (Fig. 3). On the other hand, the structure of the spermatheca, gonocoxae, ventrite VIII and extended internal part of male abdominal ventrite V in the male are very similar (these structures of *Sikkimia rufa* as in Figs 5–8, and for the other Taiwanese species as in Figs 21–23, 48, 49, 55–57, 68–70, 81–83).

**Figures 3–8.**
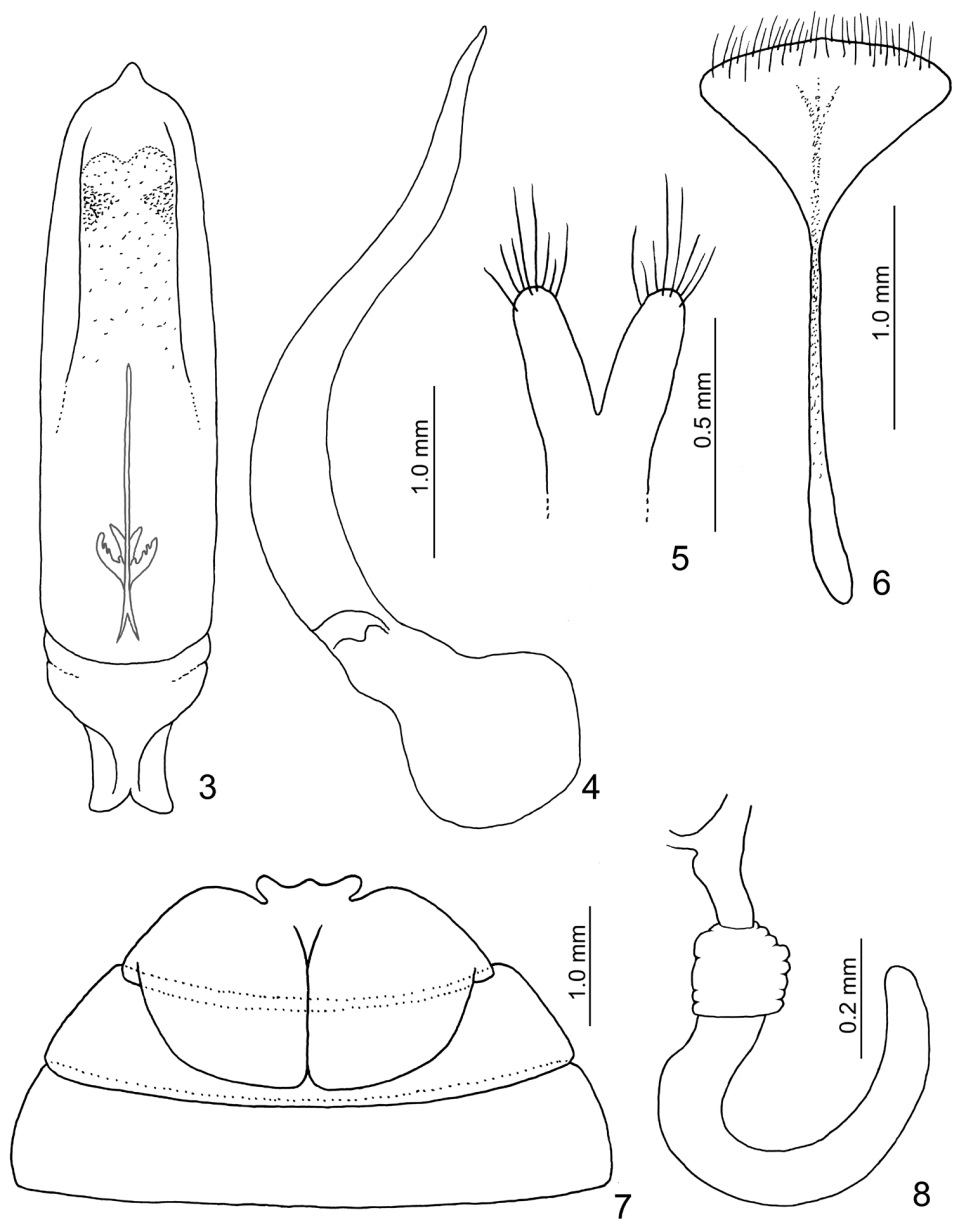
*Sikkimia rufa* (Chen). **3** Aedeagus, dorsal view **4** Aedeagus, lateral view **5** Apices of gonocoxae **6** Eigth abdominal ventrite **7** Male abdominal ventrites III–V **8** Spermatheca.

#### Biology.

Taiwanese species of *Sikkimia* appear to be univoltine, based on field observations. Larvae are nocturnal and found on the underside of the host plant’s leaves between February and April. Larval development takes about 20–22 days, based on laboratory rearing. Mature larvae leave the host plant and burrow into the soil where they build underground chambers for pupation. The pupal stage lasts for 22 days, and adults begin to emerge after April. The adults are also nocturnal and live for more than three months, a lengthy longevity for chrysomelids. Females deposited single eggs on leaves under laboratory conditions, but these failed to hatch. Presumably *Sikkimia* species overwinter as adults, as some females were collected during winter.

**Figures 9–16.**
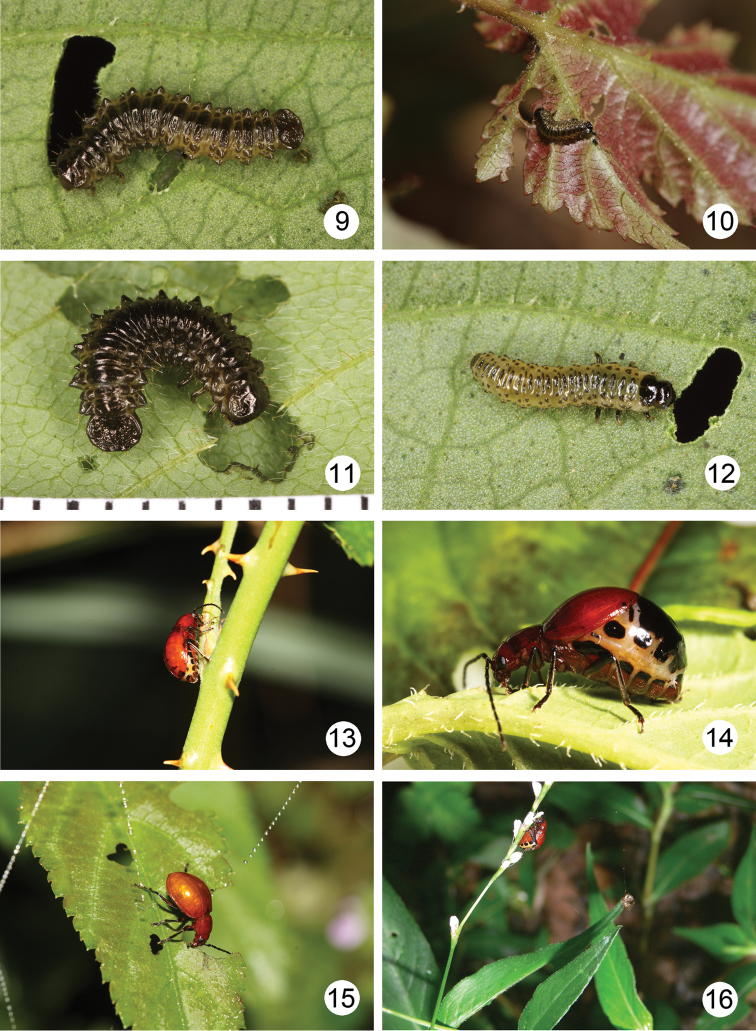
Field photography. **9** Larva of *Sikkimia sufangae* sp. n. feeding on *Polygonum chinense* **10** Larva of *Sikkimia tsoui* sp. n. feeding on *Rubus corchorifolius* **11** Larva of *Sikkimia tsoui* sp. n. feeding on Dumasia miaoliensis subsp. bicolor **12** Larva of *Gallerucida singularis* feeding on *Polygonum chinense* **13** Female of *Sikkimia tsoui* sp. n. feeding on stem of *Rubus corchorifolius* **14** Female of *Sikkimia sufangae* sp. n. **15** Male of *Sikkimia tsoui* sp. n. feeding on leaves of *Rubus swinhoei* **16** Female of *Sikkimia sufangae* sp. n. feeding on flowers of *Polygonum posumbu*.

All known *Sikkimia* species feed on the leaves of *Polygonum chinense* L. (Polygonaceae) (Fig. 9). However, some populations of *Sikkimia tsoui* sp. n. also feed on other plants in different areas. For example, populations from Yangminshan National Park (including Hsiaoyuken, Erhtzuping, Lengshuiken, Tatunshan) have been observed feeding on *Rubus swinhoei* (Fig. 15) and *Rubus corchorifolius* (Fig. 10) (Rosaceae), and members of populations from Tahunshan feed on Dumasia miaoliensis subsp. bicolor (Fig. 11) (Fabaceae). Specimens from these populations will feed on *Polygonum chinense* if switched from their original host plant.

In Taiwan, leaf beetles from three genera are known to feed on *Polygonum chinense*. These include *Altica birmanensis* (Jacoby, 1896) (Lee and Cheng 2007), *Gallerucida singularis* Harold, 1880 (Lee and Bezděk 2013), and *Sikkimia* species. *Altica birmanensis* inhabits lowlands, at elevations below 1200 m. *Gallerucida singularis* occurs at slightly higher elevations, ranging between 1000 m and 1500 m. *Sikkimia* species occupy the higher elevations, and are found from 1000 m to 2500 m in central and southern Taiwan. Thus *Gallerucida singularis* is sympatric with *Sikkimia* species in some areas. Although members of both taxa are nocturnal, their larvae prefer different sites on the host plant. Larvae of *Gallerucida singularis* always appear on the upper surface of leaves, their body segments lack lateral expansions, and the apical posterior tergites are narrower (Fig. 12). *Sikkimia* larvae occur on the underside of leaves, each body segment has lateral expansions, and the apical posterior tergites are wider (Figs 9–11).

#### Distribution.

China, India (Sikkim), Laos, Myanmar, Taiwan, and Vietnam.

### Revision of Taiwanese *Sikkimia*

#### Sikkimia babai

Taxon classificationAnimaliaColeopteraChrysomelidae

(Kimoto, 1989) comb. n.

Taiwanolepta babai Kimoto, 1989: 74.

##### Type locality.

Taiwan: Kaoshiung county, Shinanshan (溪南山), 23°05'36"N, 120°48'18"E, 2600 m.

##### Type material.

Deposition of type specimens (holotype and one paratype) was not indicated by the original paper. The paratype ♂was found at the KMNH, labeled: “Thu Yun Shan [出雲山], near Liu Kui [六龜], S-Taiwan 23.VII.1986 Col. K Baba / *Taiwanolepta babai* n. sp. Det. S. Kimoto, 1989 / PARATYPE (printed on blue paper) / PHOTO (printed on red paper)”.

##### Other material examined

(n= 18). **Kaoshiung**: 7♂♂, 7♀♀, Tengchi (藤枝), 23°04'02"N, 120°45'21"E, 2.VI.2008, leg. C.-F. Lee (2 spec. in JBCB); 1♂, same locality, 26.V.2009, leg. C.-F. Lee; 1♀, Shihshan logging trail (石山林道, =Tengchi), 1.X.2008, leg. M.-H. Tsao; 1♂, 3♀♀, same locality, 2.X.2008, leg. M.-H. Tsou.

##### Description

**Figures 17–23.**
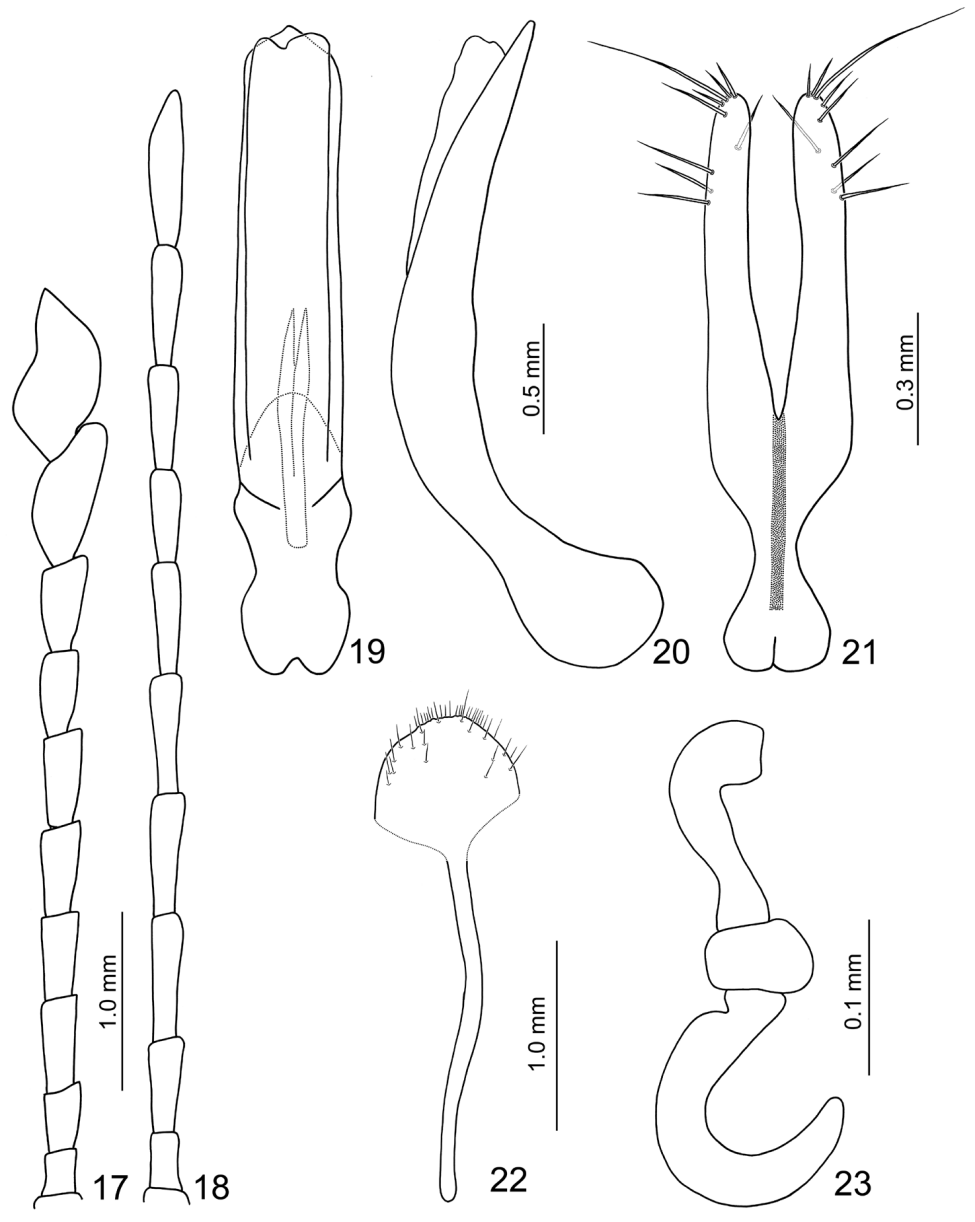
*Sikkimia babai* (Kimoto). **17** Antenna, male **18** Antenna, female **19** Aedeagus, dorsal view **20** Aedeagus, lateral view **21** Gonocoxae **22** Eighth abdominal ventrite **23** Spermatheca.

**Figures 24–35.**
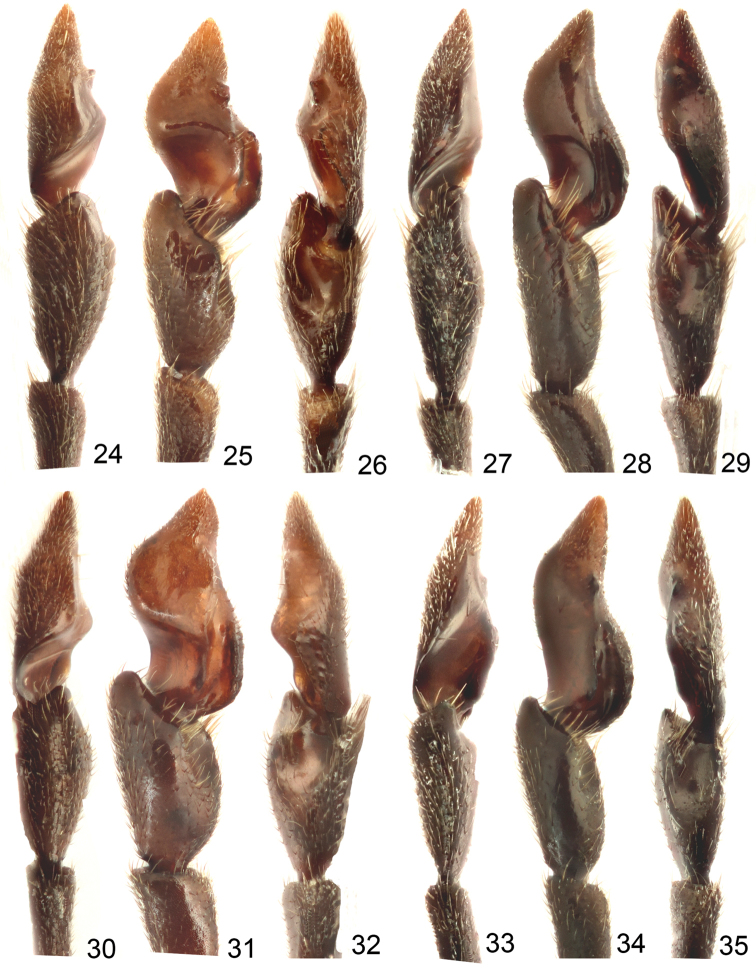
Photographs of male antennomeres X–XI. **24** *Sikkimia babai* (Kimoto), outer view **25** Ventral view **26** Inner view **27** *Sikkimia meihuai* sp. n., outer view **28** Ventral view **29** Inner view **30** *Sikkimia sufangae* sp. n., outer view **31** Ventral view **32** Inner view **33** *Sikkimia yuae* sp. n., outer view **34** Ventral view **35** Ditto, inner view.

**Figures 36–47.**
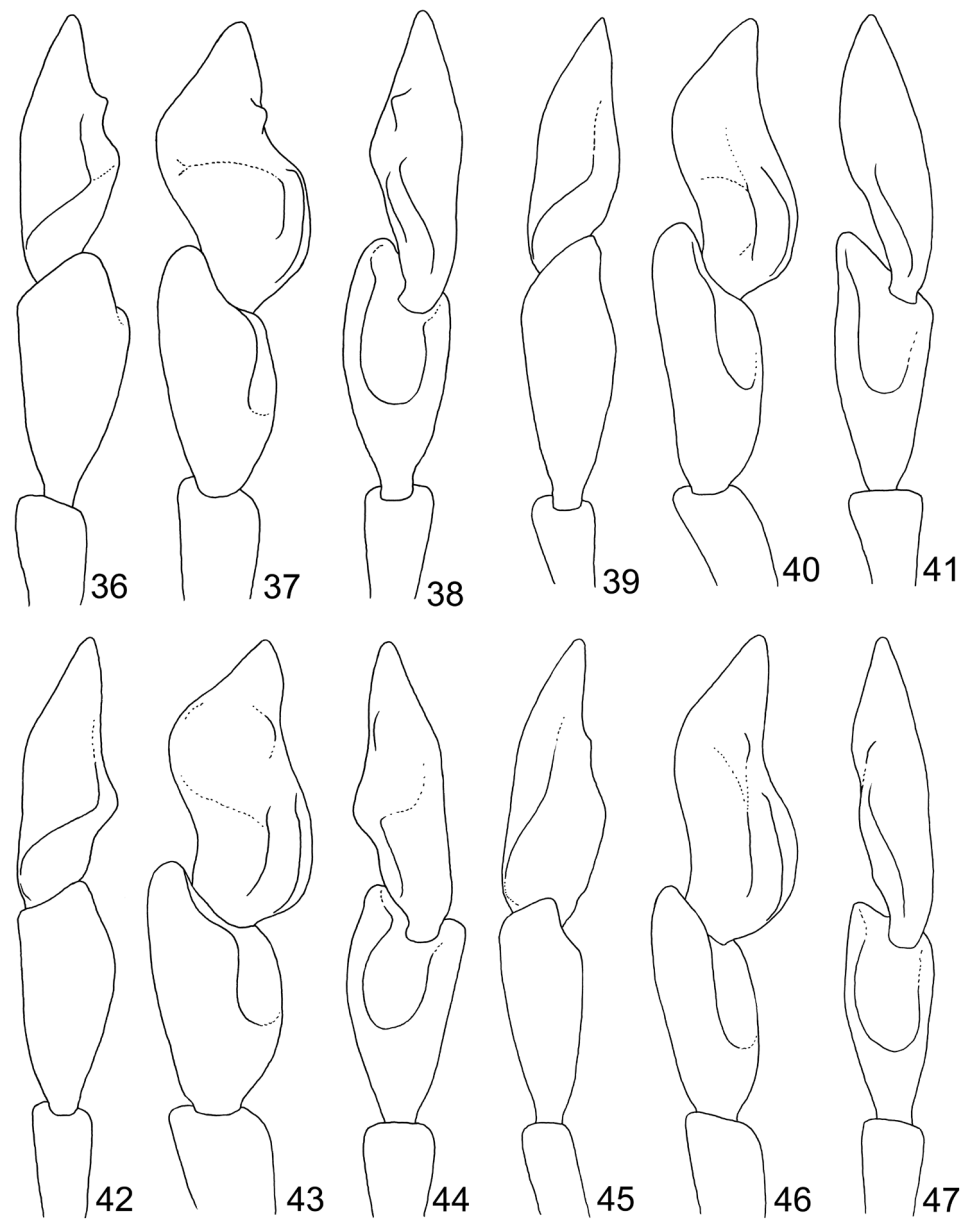
Illustrations of male antennomeres X–XI. **36** *Sikkimia babai* (Kimoto), outer view **37** Ventral view **38** Inner view **39** *Sikkimia meihuai* sp. n., outer view **40** Ventral view **41** Inner view **42** *Sikkimia sufangae* sp. n., outer view **43** Ventral view **44** Inner view **45** *Sikkimia yuae* sp. n., outer view **46** Ventral view **47** Inner view.

**Figures 48–49.**
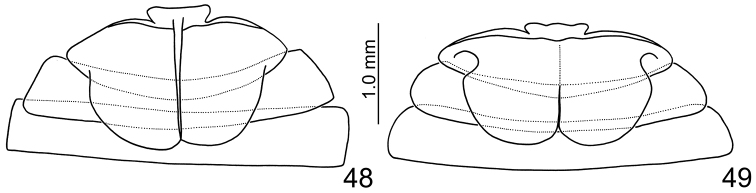
Male abdominal ventrites III–V, dorsal view. **48** *Sikkimia babai* **49** *Sikkimia tsoui* sp. n.

**Male.** Length 7.1–7.5 mm; width 3.9–4.1 mm. Coloration reddish-brown, head dark brown, legs and antennae black. Antenna (Fig. 17) elongate, about as long as body; antennomeres I to VIII filiform; IX widening slightly towards apex; × and XI extremely swollen (Figs 24–26, 36–38), × with a deep groove, from middle to apex, of mesal surface; apex of XI pointed, weakly concave in apical 1/3 of mesal surface and in basal 1/4 of outer surface; dorsal surface with two longitudinal ridges, one centrally located, curved, from middle to basal 1/5; other longitudinal ridge along mesal surface from middle to basal 1/4; one deep groove between the two longitudinal ridges; one transverse groove near base; small process at apical 1/3 near outer margin; length ratios of antennomeres II to XI about 1.0 : 1.2 : 2.0 : 1.9 : 1.9 : 1.9 : 1.7 : 1.9 : 3.2 : 3.5, and length to width ratios of antennomeres II to XI about 1.4 : 1.5 : 2.4 : 2.1 : 2.1 : 2.3 : 2.2 : 1.8 : 2.4 : 2.0. Pronotum transverse, 1.7 × wider than long; anterior and posterior margins almost straight; lateral margins weakly rounded or straight; disc finely punctured. Elytra narrow, about 1.3 × longer than wide; densely and randomly punctuate, humeri reduced. Abdominal ventrite V (Fig. 48) trilobed, internal anterior margin extended, reaching ventrite III; median longitudinal, internal ridge running from base to apex of extension. Abdominal tergite I with only spiracles sclerotized; tergites II–V with sclerotized spiracles and transverse weakly sclerotized areas; most of tergite VI and spiracles strongly sclerotized; tergite VII entirely and strongly sclerotized. Aedeagus (Figs 19–20) narrow in dorsal view, about 6.2× longer than wide, parallel-sided in basal 1/3, becoming slightly narrower towards apex; apex subtriangular and pointed; ventral surface well sclerotized and smooth; narrow and moderately curved in lateral view; endophallic sclerite longitudinal and slender, bifurcate apically, about 0.3 × as long as aedeagus.

**Female.** Length 8.1–8.4 mm; width 5.3–5.8 mm. Similar to males, but dark brown ventrally; antennae (Fig. 18) filiform, antennomeres × and XI not swollen; length ratio of II to XI about 1.0 : 1.7 : 2.2 : 2.2 : 2.2 : 2.0 : 1.8 : 1.9 : 2.2 : 2.8, and length to width ratios of II to XI about 1.9 : 2.4 : 3.4 : 3.6 : 3.8 : 3.6 : 3.2 : 3.3 : 3.7 : 4.4. Elytra wider than in male, length equal to width. Gonocoxae (Fig. 21) slender, together about 4.0 × longer than wide, joined from base almost to middle, base strongly narrowed in basal 1/3 with a long medial groove, apices tubular and sub-parallel, inner margins slightly indented medially, apex with nine setae. Ventrite VIII (Fig. 22) with extremely long spiculum; apical margin widely rounded, weakly sclerotized basally, disc with long scattered setae along apical margin. Abdominal tergites I–III membranous, only spiracles sclerotized, tergites IV–VII entirely and strongly sclerotized. Receptacle of spermatheca (Fig. 23) strongly swollen and transverse, pump elongate and moderately curved, proximal spermathecal duct long and wide.

##### Diagnosis.

*Sikkimia babai* is similar to *Sikkimia sufangae* sp. n. They share a slender aedeagus (more than 5.9× longer than wide), but in *Sikkimia babai* it is parallel-sided (aedeagus wider basally in *Sikkimia sufangae* sp. n. (Fig. 66, 67)). Antennomere XI in male *Sikkimia babai* has one process on the inner antero-lateral surface and the outer antero-lateral surface is flat (process absent on inner antero-lateral surface and outer antero-lateral surface depressed in *Sikkimia sufangae* sp. n.). The gonoxae are sub-parallel in *Sikkimia babai* (diverging in *Sikkimia sufangae* sp. n.).

##### Host plant.

*Polygonum chinense* L. (Polygonaceae).

##### Distribution.

**Figure 50.**
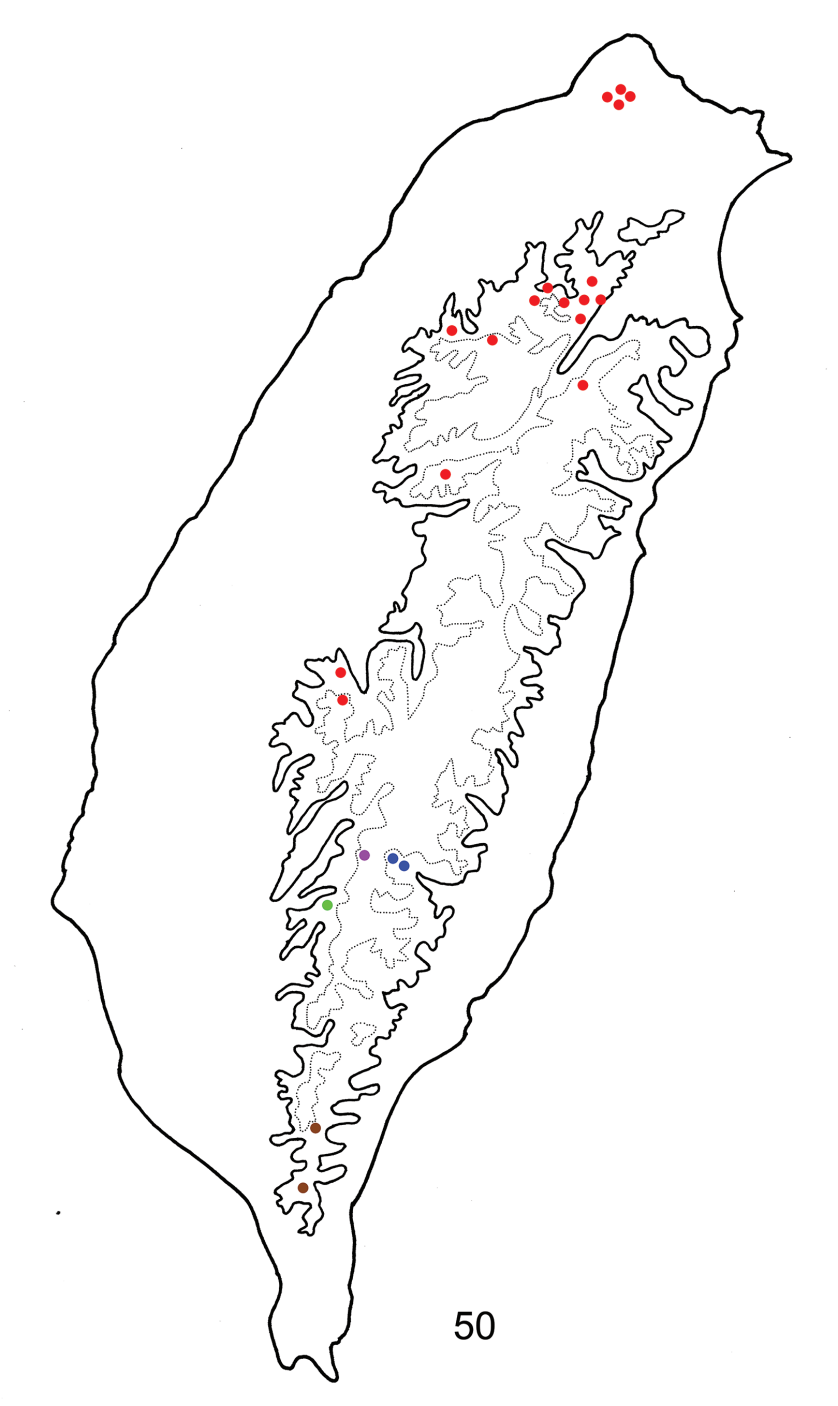
Distribution map of *Sikkimia* species of Taiwan, solid line: 1000 m, broken line: 2000 m. Brown dots: *Sikkimia sufangae* sp. n., green dots: *Sikkimia babai*, pink dots: *Sikkimia yuae* sp. n., red dots: *Sikkimia tsoui* sp. n., blue dots: *Sikkimia meihuai* sp. n.

Tengchi (Kaoshiung county) (Fig. 50) and its surrounding areas.

#### Sikkimia meihuai sp. n.

Taxon classificationAnimaliaColeopteraChrysomelidae

http://zoobank.org/DD3EE7B6-4DD2-4CD7-AAF7-F2FCD115C6CC

Figs 27–29 , 39–41 , 50 , 51–57

##### Type locality.

Taiwan: Taitung county, Liyuan (栗園), 23°13'17"N, 121°00'40"E, 1800 m.

##### Type material

(n= 19). Holotype ♂: **Taitung**: Liyuan (栗園), 23.VI.2010, leg. M.-H. Tsou. Paratypes: 3♂♂, 2♀♀, same data as holotype; 2♀♀, same locality, 19.VI.2013, leg. C.-F. Lee; 7♀♀, same locality, 24.VII.2013, leg. C.-F. Lee; 1♂, 3♀♀, Motien (摩天), 23°11'41"N, 121°01'18"E, 20.VI.2011, leg. C.-F. Lee (1♂, 2♀♀ in JBCB).

##### Description

**Figures 51–57.**
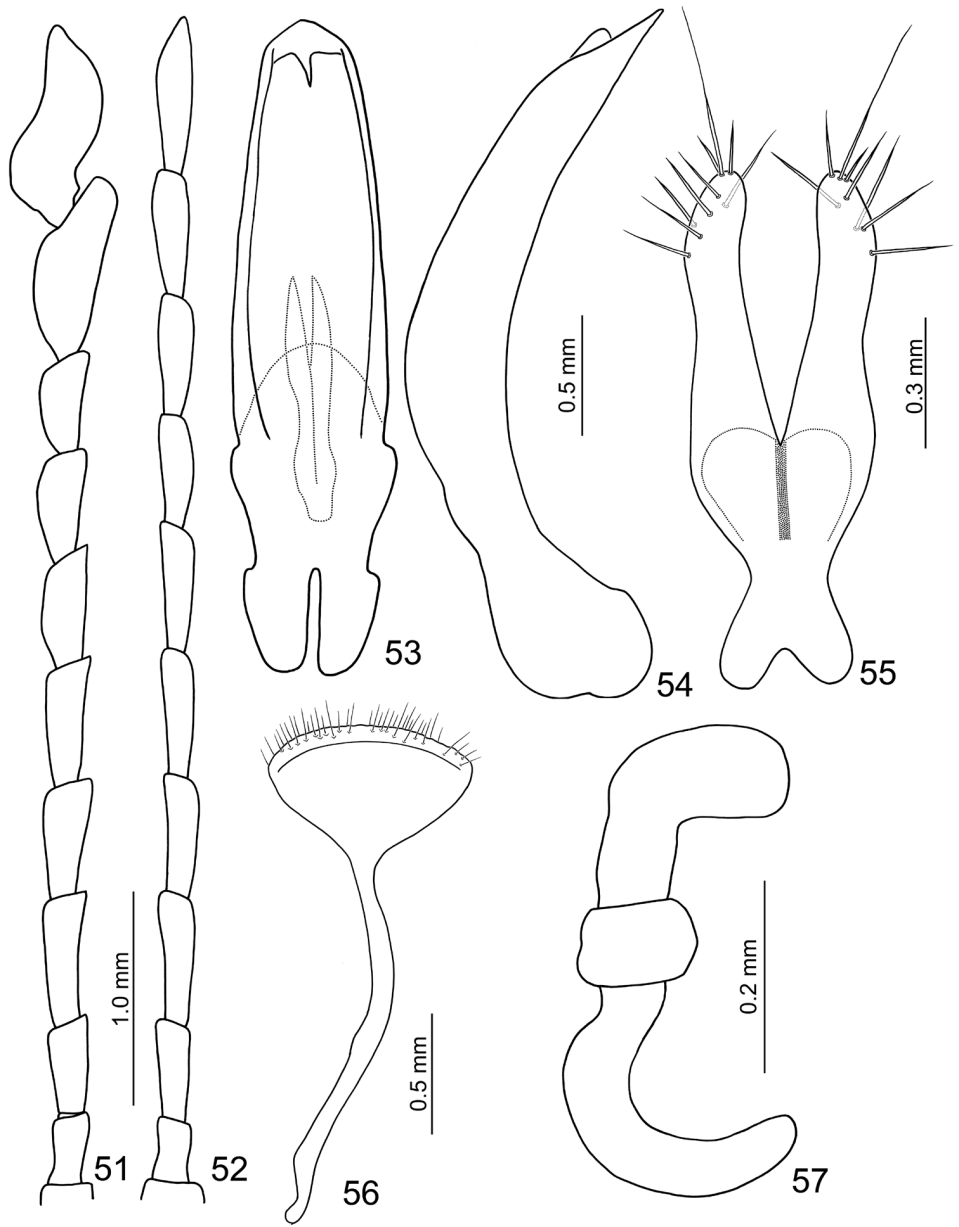
*Sikkimia meihuai* sp. n. **51** Antenna, male **52** Antenna, female **53** Aedeagus, dorsal view **54** Aedeagus, lateral view **55** Gonocoxae **56** Eighth abdominal ventrite **57** Spermatheca.

**Male.** Length 7.3–7.5 mm; width 4.0–4.2 mm. Coloration brown, head dark brown, legs and antennae black. Antenna (Fig. 51) long, about as long as body; antennomeres I–VII filiform; VIII-IX widening slightly;× and XI extremely swollen (Figs 27–29, 39–41),× with a shallow groove from middle to apex of mesal surface, XI moderately concave in basal 1/4 of outer surface, weakly concave in apical 1/3 of mesal surface and pointed apically; dorsal surface with two longitudinal ridges, one close to mesal margin, extending from middle and abbreviated near base, other longitudinal ridge along mesal margin extending from apical 1/3, projecting in middle, and ending in basal 1/4, with a deep groove between the longitudinal ridges, and a transverse groove near the base; length ratio of II to XI about 1.0 : 1.3 : 1.8 : 1.6 : 1.8 : 1.6 : 1.4 : 1.3 : 1.8 : 2.8, and length to width ratios of II to XI about 2.0 : 2.0 : 2.7 : 2.4 : 2.7 : 2.2 : 2.3 : 1.9 : 2.8 : 2.8. Pronotum transverse, 1.5× as wider than long; anterior and posterior margins almost straight, slightly concave medially; lateral margin weakly rounded; disc with finely punctured. Elytra narrow, about 1.2× longer than wide; densely and randomly punctuate, humeri reduced. Abdominal ventrite V trilobed, internal anterior margin extended, reaching ventrite III; median longitudinal internal ridge running from base to apex of extension. Abdominal tergite I with only spiracles sclerotized; tergites II–V with sclerotized spiracles and transverse weakly sclerotized areas; most of tergite VI and spiracles strongly sclerotized; tergite VII entirely and strongly sclerotized. Aedeagus (Figs 53–54) wide in dorsal view, about 4.8× longer than wide, base strongly incised medially, wide in basal 1/3, becoming slightly narrower towards the subtriangular apex; ventral surface well sclerotized and smooth; broad and moderately curved in lateral view; endophallic sclerite longitudinal and slender, bifurcate apically, about 0.3× as long as aedeagus.

**Female.** Length 7.5–8.2 mm; width 4.5–4.8 mm. Similar to male, but antennae (Fig. 52) filiform, antennomeres× and XI not swollen; length ratio of II to XI about 1.0 : 1.6 : 2.1 : 2.0 : 2.1 : 2.0 : 1.8 : 1.9 : 2.1 : 2.6, and length to width ratios of II to XI about 1.9 : 2.6 : 3.3 : 3.1 : 3.3 : 3.2 : 2.9 : 3.0 : 3.3 : 4.2. Elytra relatively wide, about 1.1× longer than wide. Gonocoxae (Fig. 55) wide, together about 2.7× longer than wide and joined from base almost to middle, basal margin deeply indented medially narrowing strongly in basal 1/3 with a short medial groove, apices tubular and parallel, narrowing slightly in apical 1/3 and curving inward, with nine setae. Ventrite VIII (Fig. 56) weakly sclerotized; with extremely long speculum; apex transverse, apical margin widely rounded, with scattered long setae along apical margin. Abdominal tergites I–III membranous with only spiracles sclerotized, tergites IV–VII entirely and strongly sclerotized. Receptacle of spermatheca (Fig. 57) strongly swollen and transverse; pump elongate and strongly curved; spermathecal duct short but extremely wide.

##### Diagnosis.

*Sikkimia meihuai* sp. n. is similar to *Sikkimia yuae* sp. n. in greatest width of the aedeagus (4.8× longer than wide), but differs in having the aedeagus narrowing very slightly towards the apex (distinctly narrower in apical 1/3 in *Sikkimia yuae* sp. n.); short median ridge on antennomere IX in males (long median ridge in *Sikkimia yuae* sp. n.); and wider gonocoxae, 2.7× longer than wide (slender gonocoxae in *Sikkimia yuae* sp. n., 4.4× longer than wide).

##### Host plant.

*Polygonum chinense* L. (Polygonaceae).

##### Etymology.

This new species is named after Mr. Mei-Hua Tsou, who is a member of TCRT and the first to collect this new species.

##### Distribution.

East half of South Cross-Island Highway (南橫公路) (Fig. 50).

#### Sikkimia sufangae sp. n.

Taxon classificationAnimaliaColeopteraChrysomelidae

http://zoobank.org/9AD84610-CAB7-4517-934E-689E4A8F3393

Figs 9 , 14 , 16 , 30–32 , 42–44 , 50 , 58–63 , 64–70

##### Type locality.

Taiwan: Pingtung county, Tahanshan (大漢山), 22°24'27"N, 120°45'23"E, 1400 m.

##### Type material

(n= 75). Holotype ♂: **Pingtung**: Tahanshan (大漢山), 6.VI.2012, leg. C.-F. Lee. Paratypes: 5♂♂, 9♀♀, same data as holotype (2♂♂, 2♀♀ in JBCB); 1♂, same locality, 18.VII.2007, leg. C.-F. Lee; 1♂, 1♀, same locality, 22.I.2009; leg. S.-F. Yu; 1♂, same locality, 25.V.2009, leg. M.-L. Jeng; 1♀, same locality, 21.I.2012, leg. S.-F. Yu; 1♀, same locality, 19.VII.2012, leg. C.-F. Lee; 2♂♂, same locality, 29.IV.2013, leg. Y.-T. Chung; 1♂, same locality, 2.VI.2013, leg. J. Yamasako (EUMJ); 1♀, same locality, 29.VI.2013, leg. B.-X. Guo; 3♀♀, same locality, 8.VII.2013, leg. B.-X. Guo; 3♀♀, same locality, 11.VII.2013, leg. B.-X. Guo; 1♀, same locality, 12.VII.2013, leg. Y.-T. Chung; 1♀, same locality, 19.VII.2013, leg. M.-H. Tsou; 1♀, 28.VIII.2014, leg. Y.-T. Chung; 3♀♀, 4.X.2014, leg. Y.-T. Chung; 8♂♂, 1♀, same locality, 1.V.2015, leg. Guo & Chung; 1♂, same locality, 19.V.2015, leg. Y.-T. Chung; 3♂♂, same locality, 27.V.2015, leg. Y.-T. Chung; 1♂, same locality, 29.V.2015, leg. Y.-T. Chung; 4♂♂, 3♀♀, same locality, 6.VI.2015, leg. Y.-T. Chung; 3♂♂, Peitawushan (北大武山), 22°37'47"N, 120°45'41"E, 22.IV.2015, leg. J.-C. Chen; 7♂♂, 7♀♀, same locality, 24.IV.2015, leg. J.-C. Chen.

##### Description

**Figures 58–63.**
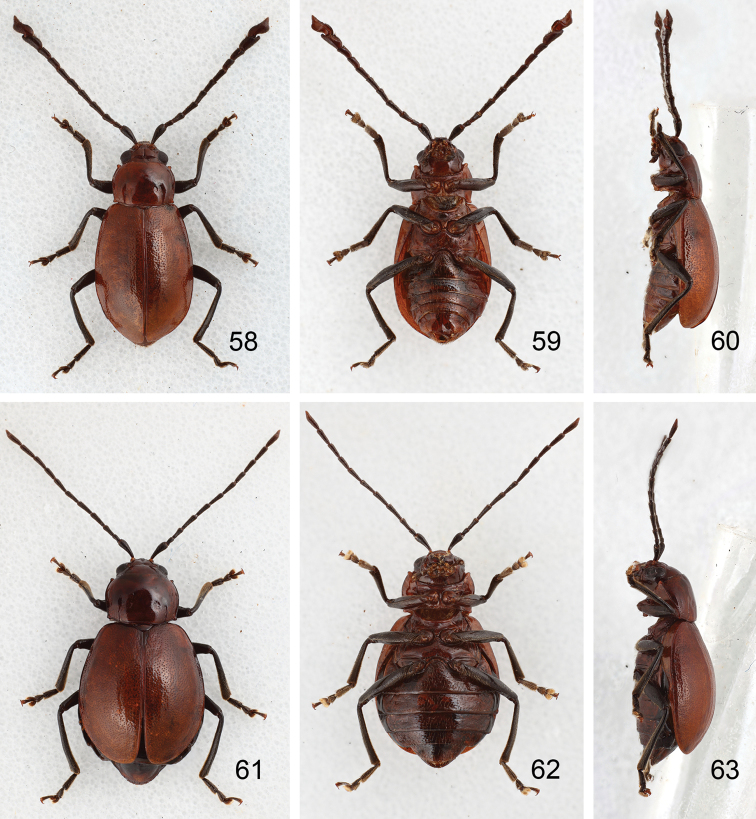
Habitus of *Sikkimia sufangae* sp. n. **58** Male, dorsal view **59** Male, ventral view **60** Male, lateral view **61** Female, dorsal view **62** Female, ventral view **63** Female, lateral view.

**Figures 64–70.**
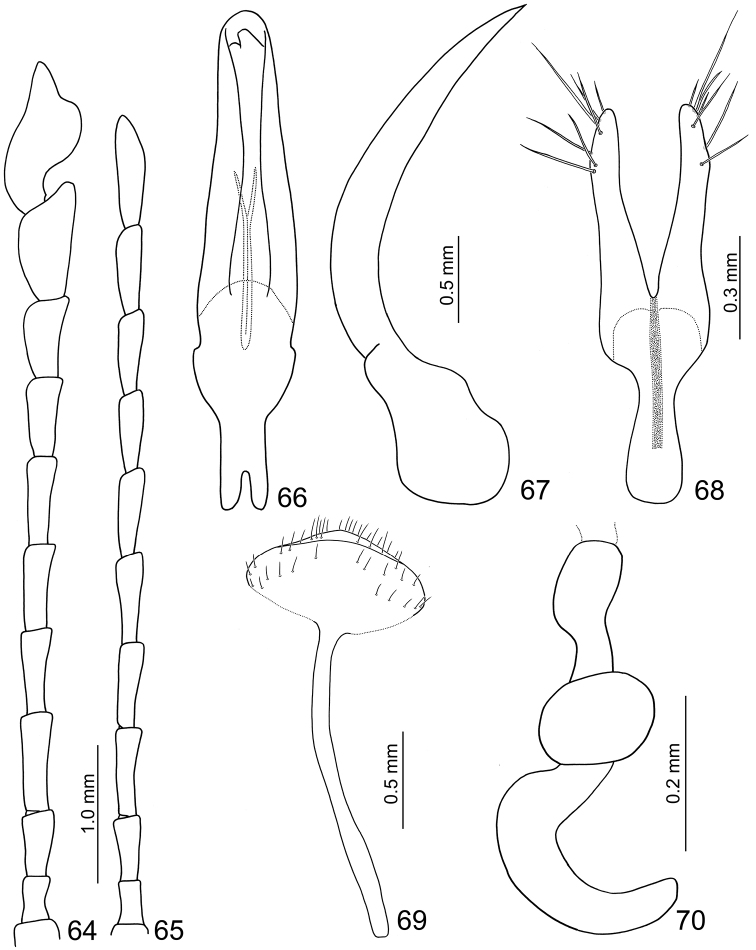
*Sikkimia sufangae* sp. n. **64** Antenna, male **65** Antenna, female **66** Aedeagus, dorsal view **67** Aedeagus, lateral view **68** Gonocoxae **69** Eighth abdominal ventrite **70** Spermatheca.

**Male.** Length 7.8–9.0 mm; width 4.0–4.3 mm. Coloration (Figs 58–60) brown, head dark brown, legs and antennae black. Antenna (Fig. 64) long, about as long as body; antennomeres I to VII filiform; VIII and IX widening slightly;× and XI (Figs 30–32, 42–44) extremely swollen,× with shallow groove from middle to apex of mesal surface; apex of XI pointed, moderately concave in basal 1/4 of outer surface and in apical 1/3 of mesal surface, dorsal surface with two longitudinal ridges, one close to mesal margin, from base to near middle, strongly curved; other longitudinal ridge along mesal margin extending from basal ¼ to apical 1/3, projecting medially, with a deep groove between the longitudinal ridges, and a transverse groove near the base, shallowly depressed on outer antero-lateral surface; length ratio of II to XI about 1.0 : 1.4 : 1.8 : 2.2 : 2.0 : 1.9 : 2.1 : 1.9 : 1.8 : 2.8, and length to width ratios of II to XI about 1.6 : 1.9 : 2.9 : 2.7 : 2.6 : 2.8 : 2.8 : 2.5 : 1.9 : 2.2. Pronotum transverse, 1.5× wider than long; anterior and posterior margins almost straight; lateral margin weakly rounded; disc with small punctures. Elytra narrow, about 1.4× longer than wide; densely and randomly punctuate, humeri reduced. Abdominal ventrite V trilobed, internal anterior margin extended, reaching ventrite III; median longitudinal, internal ridge running from base to apex of extension. Abdominal tergite I with only spiracles sclerotized; tergites II-V with sclerotized spiracles and transverse weakly sclerotized areas; most of tergite VI and spiracles strongly sclerotized; tergite VII entirely and strongly sclerotized. Aedeagus (Figs 66–67) slender, about 5.9× longer than wide, base moderately incised medially, basal 1/3 wide, narrowing considerably towards the rounded apex, ventral surface well sclerotized and smooth; moderately curved in lateral view; endophallic sclerite longitudinal and extremely slender, bifurcate apically, about 0.3× as long as aedeagus.

**Female.** Length 7.8–8.1 mm; width 5.2–5.3 mm. Similar to males (Figs 61–63), but antennae filiform (Fig. 65), antennomeres× and XI not swollen; length ratio of II to XI about 1.0 : 1.5 : 2.0 : 1.9 : 2.0 : 1.9 : 1.8 : 1.9 : 2.0 : 2.6, and length to width ratios of II to XI about 1.9 : 2.3 : 3.3 : 3.2 : 3.4 : 3.1 : 3.0 : 3.1 : 3.4 : 4.0. Elytra as wide as long. Gonocoxae (Fig. 68) slender, about 3.6× longer than wide, joined from base to middle, base rounded, strongly narrowed in basal 1/3, apices tubular curved slightly inwards, diverging, apex with seven or eight setae. Ventrite VIII (Fig. 69) weakly sclerotized; apex extremely transverse, apical margin widely rounded, disc with long scattered long setae towards apex. Abdominal tergites I–III membranous with only the spiracles sclerotized, IV–VII entirely and strongly sclerotized. Receptacle of spermatheca (Fig. 70) strongly swollen and transverse, pump long and strongly curved; proximal spermathecal duct short and swollen distally.

##### Diagnosis.

*Sikkimia sufangae* is similar to *Sikkimia babai*. See diagnosis of *Sikkimia babai* for a summary of the differentiating characteristics of these two species.

##### Host plant.

*Polygonum chinense* L.; *Polygonum posumbu* Buch.-Ham. ex Don (Polygonaceae) (Fig. 16).

##### Etymology.

This new species is named after Mrs. Su-Fang Yu, who is a member of TCRT and the first to collect this new species.

##### Distribution.

Southern Taiwan (Fig. 50).

#### Sikkimia tsoui sp. n.

Taxon classificationAnimaliaColeopteraChrysomelidae

http://zoobank.org/385E17EE-64F8-4B24-AF8B-E7D2C948731C

Figs 10 , 11 , 13 , 15 , 49 , 50 , 71–76 , 77–83

##### Type locality.

Taiwan: Taipei city, Hsiaoyuken (小油坑), 25°10'38"N, 121°32'50"E, 800 m.

##### Type material

(n= 229). Holotype ♂: **Taipei**: Hsiaoyuken (小油坑), 22.VI.2008, leg. M.-H. Tsou. Paratypes: 1♀, same as holotype; 1♂, same locality, 21.IV.2008, leg. M.-H. Tsou; 2♂♂, same locality, 24.IV.2008, leg. M.-H. Tsou; 6♀♀, same locality, 22.VI.2008, leg. S.-F. Yu; 1♂, same locality, 24.V.2008, leg. M.-H. Tsou; 1♂, 3♀♀, same locality, 5.IV.2009, leg. M.-H. Tsou; 8♂♂, 13♀♀, same locality, 8.V.2010, leg. M.-H. Tsou; 2♂♂, 8♀♀, same locality, 15.V.2011, leg. M.-H. Tsou; 1♀, Erhtzuping (二子坪), 25°11'01"N, 121°31'07"E, 14.VIII.2011, leg. M.-H. Tsou; 7♂♂, 4♀♀, same locality, 3.VI.2011, leg. M.-H. Tsou; 2♀♀, Lengshuiken (冷水坑), 25°10'03"N, 121°33'46"E, 07.IV.2009, leg. H. Lee; 1♂, same locality, 08.IV.2009, leg. H. Lee; 1♀, Tatunshan (大屯山), 25°11'12"N, 121°31'22"E, 22.V.2010, leg. M.-H. Tsou; **Hsinchu**: 1♀, Lupi (魯壁), 24°39'56"N, 121°16'47"E, 19.VII.2008, leg. M.-H. Tsou; 1♂, 2♀♀, Mamei (馬美), 24°40'13"N, 121°19'13"E, 10.VII.2010, leg. M.-H. Tsou; 1♂, Tahunshan (大混山), 24°41'20"N, 121°16'29"E, 08.IV.2009, leg. M.-H. Tsou; 1♂, same locality, 11.IV.2009, leg. M.-H. Tsou; 1♀, same locality, 13.IV.2009, leg. M.-H. Tsou; 1♂, Talu logging trail (大鹿林道), 24°32'06"N, 121°07'01"E, 1.VIII.2015, leg. Y.-L. Lin; **Ilan**: 1♀, Mingchi (明池), 24°39'01"N, 121°28'22"E, 2.VII.2008, leg. H.-J. Chen; 1♂, Taipingshan (太平山), 24°29'53"N, 121°32'06"E, 5.VIII.2015, leg. Y.-T. Chung; 16♀♀, Yuanyanghu (鴛鴦湖), 24°34'36"N, 121°24'09"E, 22.VIII.2011, leg. C.-F. Lee; 10♂♂, 5♀♀, same locality, 22.VIII.2011, leg. M.-H. Tsou (2♂♂, 2♀♀ in JBCB); 15♂♂, 5♀♀, same locality, 22.VIII.2011, leg. H. Lee; 3♂♂, Tatung (大同, = Yuanyanghu), 19.VIII.2010, leg. H.-H. Lee; **Miaoli**: 1♂, Luchang (鹿場), 24°32'26"N, 121°01'38"E, 1.VI.2014, leg. Y.-M. Weng; **Nantou**: 15♂♂, 35♀♀, Hsitou (溪頭), 23°40'20"N, 120°47'53"E, 14.VI.2011, leg. C.-F. Lee; 10♂♂, 7♀♀, same locality, 9.VIII.2011, leg. M.-H. Tsou; 1♂, Shanlinhsi (杉林溪), 23°38'22"N, 120°47'32"E, 10.IX.2009, leg. Y.-T. Wang; 2♂♂, 3♀♀, same locality, 12.VIII.2015, leg. S.-P. Wu; **Taichung**: 7♂♂, 7♀♀, Anmashan (鞍馬山), 24°14'41"N, 120°58'30"E, 19.X.2011, leg. C.-F. Lee; 2♂♂, Tahsuehshan (大雪山, = Anmashan), 7.VI.2010, leg. C.-F. Lee; 2♀♀, same locality, 4.VI.2012, leg. J.-C. Chen; **Taoyuan**: 1♂, Hsuanyuan (萱源), 24°39'11"N, 121°24'17"E, 13.V.2010, leg. S.-F. Yu; 4♀♀, same locality, 1.VI.2010, leg. W.-T. Liu; 1♂, Lalashan (拉拉山), 24°40'47"N, 121°23'02"E, 20.IV.2008, leg. C.-F. Lee.

##### Description

**Figures 71–76.**
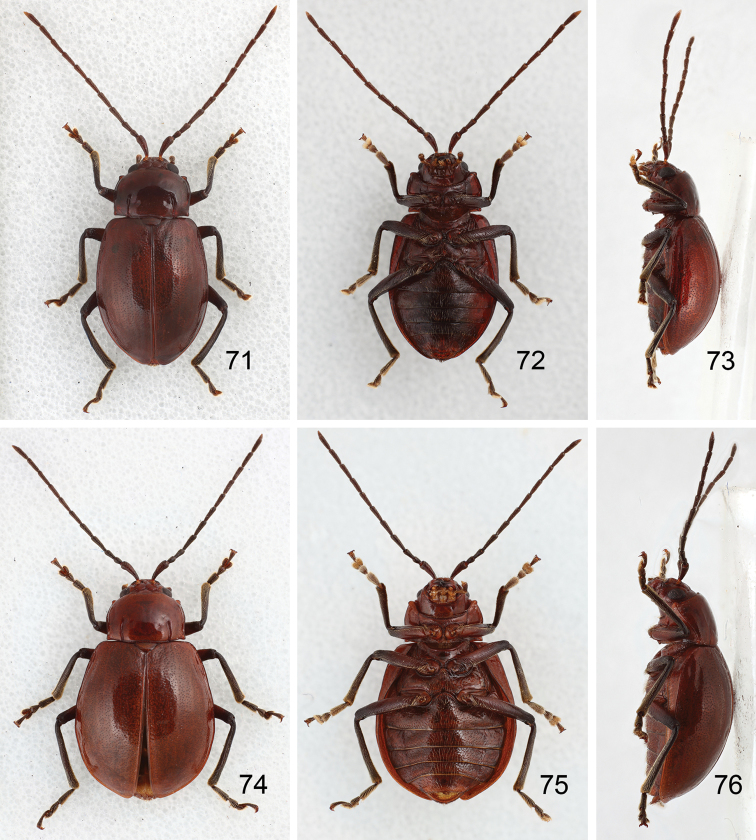
Habitus of *Sikkimia tsoui* sp. n. **71** Male, dorsal view **72** Male, ventral view **73** Male, lateral view **74** Female, dorsal view **75** Female, ventral view **76** Female, lateral view.

**Figures 77–83.**
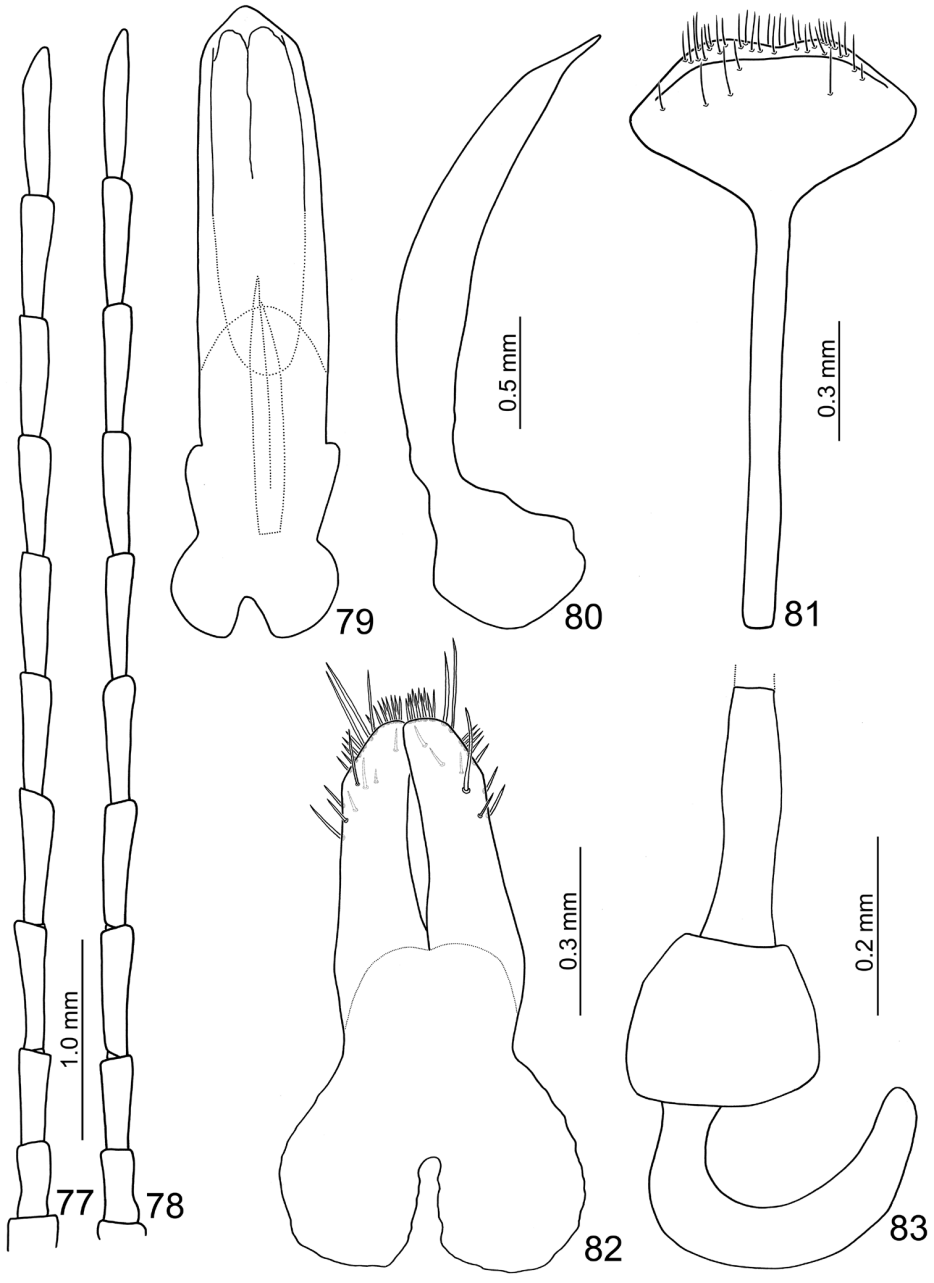
*Sikkimia tsoui* sp. n. **77** Antenna, male **78** Antenna, female **79** Aedeagus, dorsal view **80** Aedeagus, lateral view **81** Eighth abdominal ventrite **82** Gonocoxae **83** Spermatheca.

**Male.** Length 6.1–6.5 mm; width 3.7–3.8 mm. Coloration brown (Figs 71–73), legs and antennae black. Antenna (Fig. 77) long, about long as body; filiform; length ratio of antennomeres II to XI about 1.0 : 1.3 : 1.8 : 1.8 : 1.7 : 1.7 : 1.7 : 1.7 : 1.7 : 2.1 and length to width ratios of II to XI about 2.1 : 2.6 : 3.6 : 3.6 : 4.0 : 4.0 : 4.0 : 4.0 : 4.0 : 4.9. Pronotum transverse, 1.6× wider than long; anterior and posterior margins almost straight; lateral margin weakly rounded; disc with reduced punctures. Elytra wide, about 1.2× longer than wide; densely and randomly punctuate, humeri reduced. Abdominal tergite I membranous with sclerotized spiracles; II-VI with medial transverse patch sclerotized as well as area surrounding spiracles; VII mostly sclerotized, with spiracle inside sclerotized area. Abdominal ventrite V (Fig. 49) trilobed, internal anterior margin extended, reaching ventrite III; median longitudinal, internal ridge extending from base to mid-length of extension. Aedeagus (Figs 79–80) wide in dorsal view, about 4.5× longer than wide, base shallowly incised medially; greatest width in basal 1/3, becoming very slightly narrower towards the subtriangular apex; ventral surface well sclerotized, concave medially; narrow and moderately curved in lateral view; endophallic sclerite longitudinal, slender, joined from base to near apex, about 0.4× as long as aedeagus (Fig. 79).

**Female.** Length 8.0–8.3 mm; width 4.9–5.7 mm. Similar to male (Figs 74–76). Antenna (Fig. 78) about long as body; filiform; length ratio of antennomeres II to XI about 1.0 : 1.3 : 1.8 : 1.8 : 1.7 : 1.7 : 1.7 : 1.8 : 1.8 : 2.1 and length to width ratios of II to XI about 2.2 : 3.0 : 3.9 : 3.8 : 4.0 : 4.0 : 4.0 : 4.2 : 4.3 : 6.0. Elytra as long as wide, wider than in male. Gonocoxae (Fig. 82) extremely wide, about 2.9× longer than wide, joined from basal 1/5 to middle, with several long and many short dense setae on tubular apices; greatest width at base incised medially, narrowing slightly in basal 1/3 before widening slightly again. Abdominal tergites I and II membranous, only area surrounding of spiracles sclerotized; III with one pair of transverse sclerotized areas near middle; IV with one transverse sclerotized area at middle; V and VI with sclerotized areas larger than on IV; VII mostly sclerotized with spiracles lying inside sclerotized area. Ventrite VIII (Fig. 81) strongly sclerotized; apex transverse, apical margin weakly emarginate, with long dense setae along apical margin. Receptacle of spermatheca (Fig. 83) swollen, pump long and strongly curved, spermathecal duct long and slender.

##### Variation.

Specimens collected from Hsiaoyuken have more robust antennae (length ratio of antennomeres II to XI about 1.0 : 1.5 : 2.2 : 2.1 : 2.2 : 2.1 : 2.1 : 2.2 : 2.3 : 2.7 and length to width ratios of II to XI about 1.6 : 2.4 : 3.2 : 3.4 : 3.5 : 3.3 : 3.3 : 3.7 : 3.7 : 4.3).

##### Diagnosis.

This species is easily distinguished from other Taiwanese species of *Sikkimia* using a combination of the following characters: filiform antennae in males (swollen antennomeres× and XI in other species), reduced median ridge on internal anterior margin extension of abdominal ventrite V (well developed internal median ridge in other species), and the endophallic sclerite of aedeagus that is joined from the base almost to the apex (endophallic sclerite of aedeagus bifurcate apically in other species); abdominal tergites IV-VI largely membranous in female, and gonocoxae much wider with numerous setae on their apices (other species with entirely sclerotized abdominal tergites, slender gonocoxae with few setae on their apices in other species).

##### Host plant.

*Polygonum chinense* L.; *Polygonum thunbergii* Sieb. & Zucc. (Polygonaceae); *Rubus swinhoei* Hance; *Rubus corchorifolius* L. f. (Rosaceae); Dumasia miaoliensis Y. C. Liu & F. Y. Lu subsp. bicolor (Hayata) Ohashi & Tateishi (Fabaceae).

##### Etymology.

This new species is named after Mr. Mei-Hua Tsou, who is a member of TCRT and the first to collect this new species.

##### Distribution.

North and Central Taiwan (Fig. 50). The distribution extend northwards to Yamingshan National Park (陽明山國家公園) and southwards to Hsitou (溪頭).

#### Sikkimia yuae sp. n.

Taxon classificationAnimaliaColeopteraChrysomelidae

http://zoobank.org/288CCEA4-D157-43B8-A523-ED8B454EEDFF

Figs 33–35 , 50 , 84–90

##### Type locality.

Taiwan: Kaoshiung county, Chungchihkung (中之關), 23°17'10"N, 120°53'51"E, 2300 m.

##### Type material

(n= 16). Holotype ♂: **Kaoshiung**: Chungchihkung (中之關), 10.VI.2015, leg. C.-F. Lee. Paratypes: 5♂♂, 7♀♀, same data as holotype (2♂♂, 2♀♀ in JBCB); 1♂, 2♀♀, Taoyuan (桃源= Chungchihkung), 1.VII.2009, leg. S.-F. Yu.

##### Description

**Figures 84–90.**
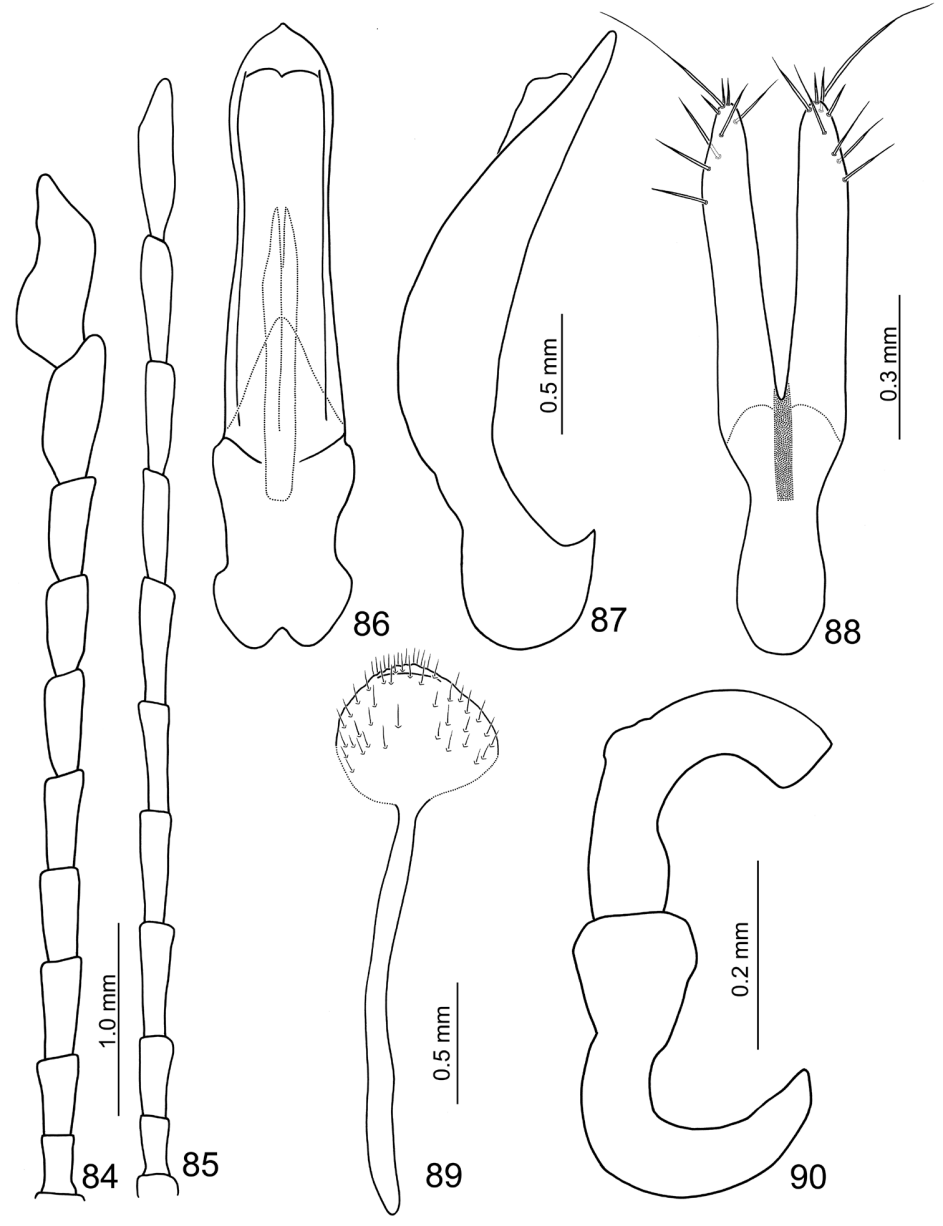
*Sikkimia yuae* sp. n. **84** Antenna, male **85** Antenna, female **86** Aedeagus, dorsal view **87** Aedeagus, lateral view **88** Gonocoxae **89** Eighth abdominal ventrite **90** Spermatheca.

**Male.** Length 7.1–7.5 mm; width 3.9–4.1 mm. Coloration reddish-brown, head dark brown, legs and antennae black. Antenna (Fig. 84) long, about long as body; antennomeres I to VII filiform; VIII and IX slightly widened;× and XI extremely swollen (Figs 33–35, 45–47),× with deep groove from middle to apex of mesal surface, apex of XI pointed, moderately concave in apical 1/3 of mesal surface and in basal 1/4 of outer surface, dorsal surface with two longitudinal ridges, one close to mesal margin extending from basal ¼ to apical 1/3, other longitudinal ridge along mesal margin from basal ¼ to the middle, deep groove between longitudinal ridges, transverse groove near base; length ratio of antennomeres II to XI about 1.0 : 1.3 : 1.5 : 1.5 : 1.6 : 1.6 : 1.5 : 1.6 : 2.5 : 3.1, length to width ratios of antennomeres II to XI about 1.8 : 1.8 : 2.4 : 2.2 : 2.3 : 2.3 : 2.1 : 2.0 : 2.7 : 2.7. Pronotum transverse, 1.6× wider than long; anterior and posterior margins sinuate, weakly concave medially; lateral bordermargin weakly rounded; disc with fine scattered punctures. Elytra narrow, about 1.2× longer than wide; punctuate densely, reduced humeri, lateral margin rounded, widest just posterior of the middle. Abdominal ventrite V trilobed, internal anterior margin extended, reaching ventrite III; median longitudinal, internal ridge running from base to apex of extension. Abdominal tergite I with only spiracles sclerotized; II-V with spiracles sclerotized and transverse weakly sclerotized areas; most of VI and spiracles strongly sclerotized; whole of VII strongly sclerotized. Aedeagus (Figs 86–87) wide in dorsal view, about 4.8× longer than wide, base shallowly incised medially; greatest width in basal 1/3, narrowing slightly towards apical 1/3, widening slightly subapically before the subtriangular apex with a pointed tip; ventral disc well sclerotized and smooth; aedeagus wide and moderately curved in lateral view; endophallic sclerite longitudinal and slender, bifurcate apically, about 0.4× as long as aedeagus.

**Female.** Length 7.8–8.2 mm; width 5.3–5.6 mm. Similar to male, but underside dark brown; antenna (Fig. 85) filiform, antennomeres× and XI not swollen; length ratio of antennomeres II to XI about 1.0 : 1.2 : 1.9 : 1.8 : 1.9 : 1.9 : 1.7 : 1.9 : 2.1 : 2.6, and length to width ratios of II to XI about 2.0 : 2.0 : 3.2 : 3.4 : 3.5 : 3.4 : 3.3 : 3.5 : 4.8 : 4.3. Elytra wider than in male, length and width the same. Gonocoxae (Fig. 88) slender, about 4.4× longer than wide, joined from base to just before middle, apices tubular, straight and subparallel, with 9–10 apical setae, base rounded and slightly narrower than greatest width at middle, narrowing slightly at basal 1/3. Ventrite VIII (Fig. 89) with extremely long spiculum; apex very small and oval in shape, weakly sclerotized basally, disc with long scattered setae. Abdominal tergites I–III membranous with only the spiracles sclerotized, IV–VII entirely and strongly sclerotized. Receptacle of spermatheca (Fig. 90) slightly swollen, pump short and moderately curved, spermathecal duct wide and long.

##### Diagnosis.

This new species can be distinguished from others by the following combination of characters: apical 1/3 of aedeagus narrowing slightly before widening slightly again subapically (aedeagus parallel in *Sikkimia babai* and widening basally in *Sikkimia sufangae* sp. n. and *Sikkimia meihuai* sp. n.), and straight subparallel apices of gonocoxae (curved apices of gonocoxae in *Sikkimia meihuai* sp. n., *Sikkimia babai*, and *Sikkimia sufangae* sp. n.).

##### Host plant.

*Polygonum chinense* L. (Polygonaceae).

##### Etymology.

This new species is named after Mrs. Su-Fang Yu, who is a member of TCRT and the first person to collect this new species.

##### Distribution.

West half of South Cross-Island Highway (南橫公路) (Fig. 50).

#### Key to species of *Sikkimia* in Taiwan

**Table:** 

1	Antenna filiform in male (Fig. 77); median longitudinal internal ridge on abdominal ventrite V reduced, extending from base to mid-length of extension (Fig. 49); endophallic sclerite of aedeagus joined from base to near apex (Fig. 79); abdominal tergites IV-VI of females membranous (Fig. 13); gonocoxae extremely wide, base much wider than medially, apex with numerous setae (Fig. 82)	***Sikkimia tsoui* sp. n.**
–	Antennomeres× and XI of males swollen (Figs 17, 51, 64, 84), median longitudinal internal ridge on abdominal ventrite V well developed, extending from base to apex of extension (Fig. 48), endophallic sclerite of aedeagus bifurcate apically (Figs 19, 53, 66, 86); abdominal tergite IV-VI of females entirely sclerotized (Fig. 14); gonocoxae slender, base subequal or narrower than in middle, apex with few setae (Figs 21, 55, 68, 88)	**2**
2	Aedeagus narrowing slightly towards apical 1/3, widening slightly subapically (Fig. 86); median anterior ridge on antennomere XI extending into apical 1/3 in males (Figs 33–35, 45–47); apices of gonocoxae straight and subparallel (Fig. 88)	***Sikkimia yuae* sp. n.**
–	Aedeagus parallel-sided or widened basally (Figs 19, 53, 66); median ridge of antennomere XI abbreviated or curved outwards at middle in males (Figs 24–32, 36–44); apex of gonocoxa curved (Figs 21, 55, 68)	**3**
3	Aedeagus slender, more than 5.9× longer than wide (Figs 19, 66); median ridge of antennomere XI curved medially in males (Figs 24–26, 30–32); gonocoxae slender, more than 3.6× longer than wide (Figs 21, 68)	**4**
–	Aedeagus wide, 4.8× longer than wide (Fig. 53); median ridge of antennomere XI abbreviated medially in males (Figs 27–29, 39–41); gonocoxae wide, about 2.7× longer than wide (Fig. 55)	***Sikkimia meihuai* sp. n.**
4	Aedeagus parallel-sided (Fig. 19); antennomere XI in males with a small process on inner antero-lateral surface and flat on outer antero-lateral surface (Figs 24–26, 36–38); gonocoxae sub-parallel (Fig. 21)	***Sikkimia babai* (Kimoto)**
–	Aedeagus wide basally (Fig. 66); antennomere XI in males without processes on inner antero-lateral area and depressed on outer antero-lateral area (Figs 30–32, 42–44); gonocoxae diverging (Fig. 68)	***Sikkimia sufangae* sp. n.**

### Species excluded from *Sikkimia*

As mentioned by Maulik (1936) and subsequently also by Bezděk and Zhang (2006), the descriptions of *Sikkimia metallica* Jacoby, 1903 and *Sikkimia tamra* Maulik, 1936 are very different to other *Sikkimia* and their position in *Sikkimia* was regarded as doubtful. In 2007, one of us (JB) examined the type specimens of both species. They are here moved from *Sikkimia* and to *Cerophysa* Chevrolat, 1836.

The reason why Jacoby (1903) classified his new species in *Sikkimia* is unknown to us. Probably he misinterpreted or overlooked some important characters like colour of uthe venter, impressions on the pronotum, or structure of the antennae. Duvivier (1891: 154) described the pronotal impressions as “présentant de chaque côté une profonde impression oblique” what probably allows some misinterpretations. The pronotum of *Sikkimia metallica* has transverse impression in the middle more or less interrupted medially. The differences in the structure of antennae Jacoby (1903) attributed to the sexual dimorphism as the specimens of *Sikkimia metallica* are females.

Maulik (1936) also did not examined true *Sikkimia antennata* as he published only the English translation of Duvivier´s description of *Sikkimia*. It is evident that he compared *Sikkimia tamra* with Jacoby´s *Sikkimia metallica* and thus mistakenly classified his species also in *Sikkimia*.

The main differencies between true *Sikkimia* species and *Sikkimia tamra* with *Sikkimia metallica* can be described as follows: true *Sikkimia* are large (6.1–12.0 mm), robust and convex species of orange, red or brown upperside, last two antennomeres in males are strongly modified (except *Sikkimia tsoui* sp. n.), pronotum with antebasal transverse impression limited on sides by short longitudinal furrows and additional longitudinal groove parallel to lateral margin and procoxal cavities closed behind. The same characters of *Sikkimia tamra* and *Sikkimia metallica* (which simultaneously allow us to transfer both species to *Cerophysa*) are: body 5.5–6.0 mm long, narrow, subparallel, flat, with upperside metallic green, antennae without modifications; pronotum with transverse impression in the middle of pronotum and procoxal cavities open behind. The structure of antennae is variable throughout *Cerophysa*. In some species one, two or three antennomeres can be modified, but never last two antennomeres.

#### Cerophysa metallica

Taxon classificationAnimaliaColeopteraChrysomelidae

(Jacoby, 1903) comb. n.

Sikkimia metallica Jacoby, 1903: 122.

##### Type locality.

Nilgiri hills.

##### Type material.

Syntype (♀, BMNH), labeled: “Nilgiri Hills (printed on white label) / 482 (handwritten on white label) / Type (printed on red label) / Sikkimia metallica Jac. (handwritten on blue label) / Andrewes Bequest B. M. 1922–221. (printed on white label)”.

#### Cerophysa tamra

Taxon classificationAnimaliaColeopteraChrysomelidae

(Maulik, 1936) comb. n.

Sikkimia tamra Maulik, 1936: 523.

##### Type locality.

Nilgiri hills.

##### Type material.

Syntype (unsexed, BMNH), labeled: “Type (printed on white round label with red collar) / Nilgiri Hills. G. F. Hampson 94–89. (printed on white label) / Sikkimia tamra M. S. Maulik TYPE 1935 (handwritten and printed on white label)”.

### Catalogue of *Sikkimia*

*Sikkimia antennata* Duvivier, 1891 Sikkim

*Sikkimia babai* (Kimoto, 1989), comb. n. Taiwan

*Sikkimia kabakovi* (Lopatin, 2003) Vietnam

*Sikkimia meihuai* sp. n. Taiwan

*Sikkimia miranda* (Lopatin, 2003) Vietnam

*Sikkimia rufa* (Chen, 1964) China (Yunnan), Laos, Myanmar

*Sikkimia sufangae* sp. n. Taiwan

*Sikkimia tsoui* sp. n. Taiwan

*Sikkimia yuae* sp. n. Taiwan

## Discussion

Lee (2015) proposed a possible cause of brachelytry of leaf beetles for tropical forest habitats. Reduction of hind wings may result from the production of physogastric females. Nocturnal behavior increases survival since natural enemies are less of a threat. Males actively search for mates. As like survival at adverse environments such as islands, deserts and alpine regions, flight is not essential at night and energy can be diverted to egg production (Beenen and Jolivet 2008). Thus, brachelytry is a predictable evolutionary trend. Although no related reports for this hypothesis, wingless chrysomelids at tropical forest habitats can be used to test. Like Taiwanese populations of *Paraplotes*, those of *Sikkimia* are nocturnal with brachelytrous females. Moreover, the elytral calli of both sexes, and hind wings of males, are reduced. Thus Taiwanese populations of *Sikkimia* support this hypothesis of brachelytry in leaf beetles.

Species richness of *Sikkimia* in Taiwan (five species) is lower than that of *Paraplotes* (ten species) (Lee 2015), possibly due to several causes. All *Sikkimia* species are allopatric on the same mountain ranges and not separated by elevation. Only one *Sikkimia* species, *Sikkimia tsoui* sp. n., occupies northern and central Taiwan, whereas five species of *Paraplotes* are recorded from the same area. In addition to its wider distribution, *Sikkimia tsoui* sp. n. is abundant in some areas. For example, there were so many adults at Hsitou (溪頭) and Yuanyanghu (鴛鴦湖) that during one night 50 adults were collected at Hsitou and 51 at Yuanyanghu. Both features may be the result of some autamorphic characters in *Sikkimia tsoui* sp. n. Males of *Sikkimia tsoui* sp. n. have no enlarged apical antennomeres, a character that may be involved in courtship behavior. Lack of this secondary sexual character may result in low speciation. The ability to feed on a wide range of host plants, weak sclerotization of abdominal tergites, and the unique shape of gonocoxae may increase the fitness of this species.

## Supplementary Material

XML Treatment for Sikkimia

XML Treatment for Sikkimia babai

XML Treatment for Sikkimia meihuai

XML Treatment for Sikkimia sufangae

XML Treatment for Sikkimia tsoui

XML Treatment for Sikkimia yuae

XML Treatment for Cerophysa metallica

XML Treatment for Cerophysa tamra
